# Homozygous *CDKN2A/B* deletions in low- and high-grade glioma: a meta-analysis of individual patient data and predictive values of *p16* immunohistochemistry testing

**DOI:** 10.1186/s40478-024-01889-7

**Published:** 2024-11-26

**Authors:** Darius Noack, Johannes Wach, Alonso Barrantes-Freer, Nils H. Nicolay, Erdem Güresir, Clemens Seidel

**Affiliations:** 1 Department of Radiation Oncology, University Leipzig Medical Center, Stephanstraße 9a, 04103 Leipzig, Germany; 2 Department of Neurosurgery, University Leipzig Medical Center, 04103 Leipzig, Germany; 3 Paul-Flechsig Institute of Neuropathology, University Leipzig Medical Center, 04103 Leipzig, Germany; 4 Comprehensive Cancer Center Central Germany (CCCG), 04103 Leipzig, Germany

**Keywords:** CDKN2A/B, Individual patient data, Glioma, Meta-analysis, Overall survival, p16

## Abstract

*CDKN2A/B* deletions are prognostically relevant in low- and high-grade gliomas. Data on this is derived from heterogeneous series, an accurate estimation of survival risk from homozygous *CDKN2A/B* deletion is missing. Besides genetic testing, *p16*-immunohistochemistry (IHC) as a less cost intensive means for indirect detection of *CDKN2A/B* alterations is possible but not validated in larger datasets. The present meta-analysis aimed to (1) reconstruct individual patient data (IPD) and estimate overall survival (OS) stratified by *CDKN2A/B* status from all literature and to (2) determine accuracy of *p16* testing for *CDKNA2/B* detection from published studies. For survival analysis according to *CDKN2A/B* status 460 records were screened, four articles with 714 participants were included. In *IDH*-wildtype (*IDH*-wt) gliomas, 57.07% harbored the deletion compared to 9.76% in *IDH*-mutant (*IDH*-mut) gliomas. Median OS of patients with *IDH*-wt gliomas and homozygous *CDKN2A/B* deletion was 13.0 months compared to 18.0 months with non-deleted *CDKN2A/B* (*p* = 0.014, Log-Rank). With homozygous deletion of *CDKN2A/B* the risk of death was increased by 1.5 (95%-CI 1.1–2.1). Median OS in patients with *IDH*-mut gliomas without *CDKN2A/B* deletion was 92.0 months compared to 40.0 months with *CDKN2A/B* deletion (*p* < 0.001, Log-Rank). *CDKN2A/B* deletions were associated with a significantly shorter OS (HR = 3.2; 95%-CI 2.2–5.5). For *p16* IHC analysis, 10 eligible studies with 1087 examined samples were included. The cut-off for retention differed between the studies. In 588/662 *p16* retained cases *CDKN2A/B* deletions was not detected, implying a negative predictive value (NPV) of *p16* staining of 88.8%. Conversely, 279/425 *p16* absent cases showed a *CDKN2A/B* deletion resulting in a positive predictive value (PPV) of 65.6%. Sensitivity of *p16* staining for *CDKN2A/B* detection was 79.0%, specificity 80.1%. Highest diagnostic accuracy of p16 IHC was reached with a cut-off of > 5% and within *IDH-*mut glioma.

**Supplementary Information:**

The online version contains supplementary material available at 10.1186/s40478-024-01889-7.

## Introduction

Malignant gliomas are the most frequent primary intracerebral neoplasms in adults [25]. Prognosis worsens with increasing World Health Organization (WHO) Grade from > 5–10 years in Grade 2 gliomas to 1–2 years in Grade 4 gliomas [21]. Within the last decade, the WHO grading system of gliomas that is historically based upon histopathologic markers of malignancy has been significantly altered and augmented by use of molecular alterations like isocitrate dehydrogenase (*IDH*)-*1/2*-mutation [5]. Genetically, the presence of *IDH1/2*-mutations defines a distinct class of diffuse adult glioma with overall improved prognosis as *IDH*-wt glioma [14]. Among *IDH*-mut gliomas the presence of homozygous cyclin-dependent kinase inhibitor 2 A/B (*CDKN2A/B*) gene deletions of the chromosome 9p21 are associated with significantly poorer prognosis [1, 2, 19, 32, 35, 40]. Due to this, homozygous *CDKN2A/B* deleted *IDH*-mut astrocytomas are classified as WHO grade 4 [39]. Data on the impact of *CDKN2A/B* on prognosis is derived from heterogenous series and accurate estimation of prognosis in case of *CDKN2A/B* deletion is difficult. Given the increasing relevance of *CDKN2A/B* deletions, reliable and cost-effective means of detections are needed. Besides genetic testing, indirect immunohistochemical testing for *p16* is feasible but little defined in its accuracy [4, 44].

The present meta-analysis had two aims: (1) to investigate the clinical prevalence of *CDKN2A/B* deletions and the impact of *CDKN2A/B* deletions on OS in all reported series of malignant gliomas and (2) to precisely assess the test accuracy of *p16* immunohistochemistry (IHC) for indirect *CDKN2A/B* detection from all reported series so far.

## Methods and materials

### Search strategy and study inclusion

To obtain eligible studies three databases (Cochrane library, PubMed and Web of Science) were screened up to September 27, 2023. The databases were screened, and English publications were retrieved by using the following MeSH terms: (1) „CDKN2A“ AND „glioma“; (2) „CDKN2B“ AND „glioma“; (3) „CDKN2A/B“ AND „glioma“. To be included in the meta-analysis studies must provide *CDKN2A/B* status and *IDH* status as well as OS data shown by Kaplan-Meier plots with corresponding number at risk tables. Studies without number at risk tables were excluded due to impossibility of reconstructing IPD.

Suitable studies for investigating the *CDKN2A/B* and *p16* concordance were explored by using the MeSH terms: (1) “p16” AND “CDKN2A” AND “glioma”; (2) “p16” AND “CDKN2B” AND “glioma” and (3) “p16” AND “CDKN2A/B” AND “glioma”. For inclusion, studies had to report the association between *p16* staining and *CDKN2A/B* status. Covidence was used to accelerate the systematic review process by importing all references from the databases. The website provides an automatic duplication detection to eliminate redundant records. Then titles, abstracts and full text were successively screened manually to extract suitable studies.

### Data extraction

Clinical and neuropathological characteristics (e.g. age, performance status, glioma type, *IDH1*-mutation status, *MGMT* status) were summarized. The extraction of IPD required Kaplan-Meier plots with given number at risk tables to use the application *DigitizeIt* (Bormann, Version 2.5.10 on MacOS, Braunschweig, Germany). No study provides the IPD in the results or supplementary material. To gather IPD, images of the Kaplan-Meier plots are imported in *DigitizeIt.* By establishing the coordinate system and pinpointing data points on the graph, the system creates record of x- and y-values that can be exported as a CSV file. After extraction, the digitized data were used to reconstruct the Kaplan-Meier curves using the web application *IPDfromKM* (Liu et al., Version 1.2.3.0, Houston, USA) to finally obtain IPD [20].

### Statistical analysis

The reconstructed IPD of all included studies were summarized for further analysis. New Kaplan-Meier plots of the pooled data were created by the R package *Survminer* with R (Version 4.3.1, Vienna, Austria). Median OS rates were constructed and Log-rank tests as well as the hazard ratios via cox regression were performed in SPSS (IBM, Version 29.0.1.1 on Windows 10). For survival analysis *CDKN2A/B* status and *IDH* status were regarded.

## Results

### Study characteristics

**Fig. 1:**
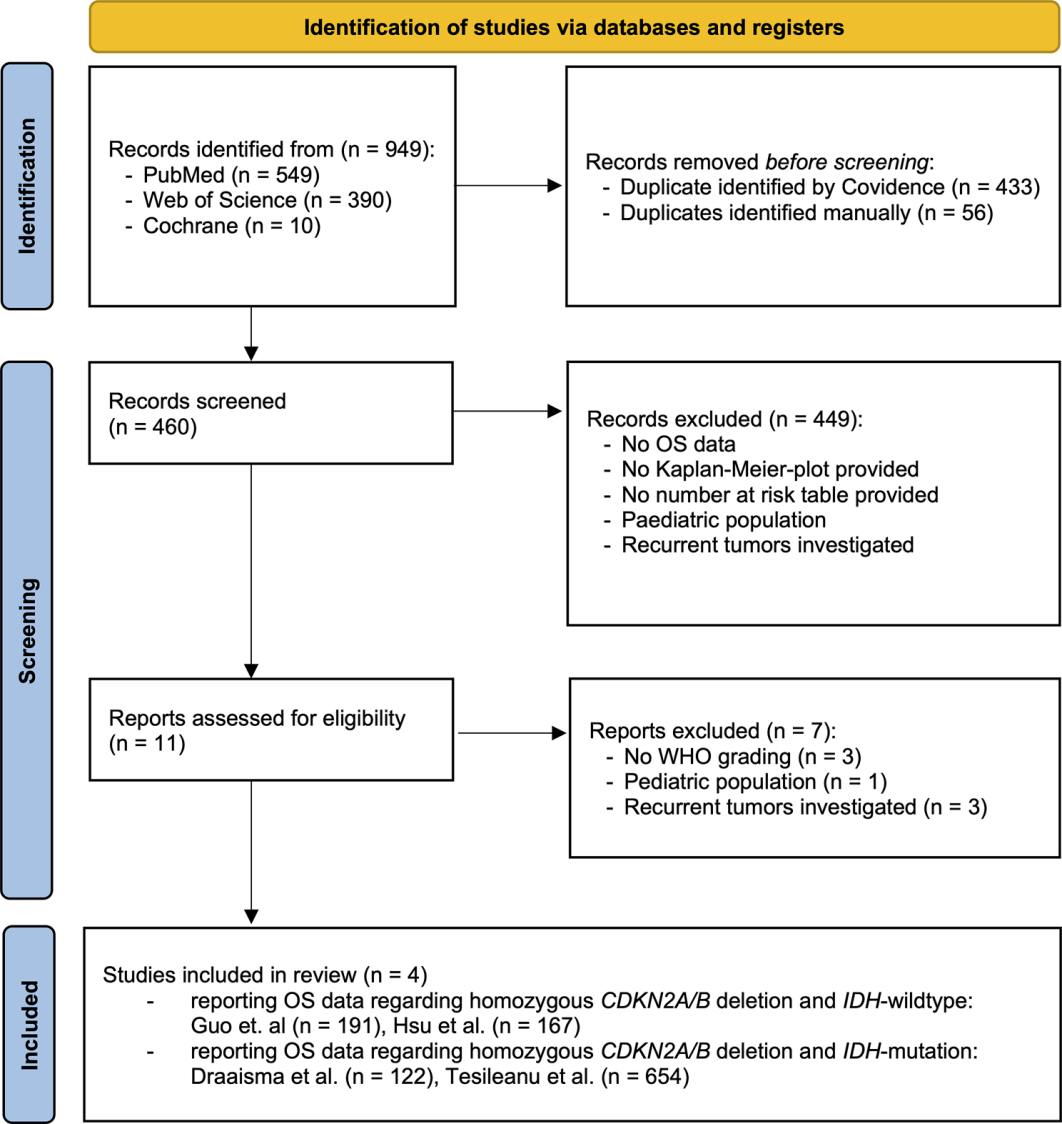
PRISMA flow diagram for study selection

The *CDKN2A/B* status and OS depicted as a Kaplan-Meier plot was provided in four eligible studies for 714 patients (Fig. 1). The included studies are authored by Draaisma et al. [9], Guo et al. [12], Hsu et al. [17] and Tesileanu et al. [40].

The results of Draaisma et al. [9] (EORTC 26091 TAVAREC Trial, phase II) and Tesileanu et al. [40] (EORTC CATNON-Trial, phase III) were generated in post-hoc analyses from randomized, multicenter studies including patients from Europe, Australia and North America. The EORTC CATNON trial primarily examined the effect of adding temozolomide (TMZ), either concurrently with radiotherapy, as adjuvant and both current and adjuvant treatment to radiotherapy in adults with newly diagnosed 1p/19q non-co-deleted anaplastic glioma [42]. Within the TAVAREC trial the use of bevacizumab (BEV) in patients with first recurrence of grade 2 or 3 gliomas who did not have 1p/19q co-deletion was assessed regarding OS [43].

The data of Guo et al. [12] and Hsu et al. [17] were compiled within monocentric retrospective analysis designed primarily for detection of influence of molecular alterations on prognosis of malignant gliomas.

### Patient characteristics

Examined tumor types differed between studies. While Hsu et al. [17] only investigated *IDH*-wt glioblastoma (GBM), Guo et al. [12] included histologic *IDH*-wt GBM and molecular *IDH*-wt GBM (histologic astrocytoma). In both studies next generation sequencing (NGS) was used to determine *CDKN2A/B* status. Hsu et al. [17] provided OS data on *CDKN2A* and *CDKN2B* separately. It was specified in the manuscript of Hsu et al. [17] that all patients harboring a *CDKN2B* deletion (*n* = 75) were also *CDKN2A* deleted, so the OS data of *CDKN2A* deleted patients (*n* = 89) were used for further investigation in the meta-analysis. Tesileanu et al. [40] analyzed patients with *IDH*-mut anaplastic astrocytoma for influence of *CDKN2A/B* deletion. Draaisma et al. [9] reported *CDKN2A/B* results on a mixed population of WHO Grade 2–4 gliomas: astrocytoma (68.6%), GBM (14.8%, not included in meta-analysis), oligodendroglioma (ODG, 2.5%, not included in meta-analysis), the histological status of the remaining samples (13.9%) was inconclusive. Both used DNA methylation profiling to examine *CDKN2A/B* status.

In the studies dealing with *IDH*-mut gliomas median patient age was lower (Tesileanu et al. [40]: 41y, range: 18–82; Draaisma et al. [9]: 43y, range: 34–52) compared to the studies of Guo et al. [12] (55.5y, range: 40.3–70.2) and Hsu et al. [17] (63y, 24.8–85.1) on *IDH*-wt GBM. Overall, more male than female patients were included (58.6% male, 41.4% female). In the study of Guo et al. [12] gender was nearly balanced (male: 52.4%) while Tesileanu et al. [40] (male 58.6%), Hsu et al. [17] (male: 60.5%) and Draaisma et al. [9] (65.6%) showed a male predominance.

Regarding follow-up data in Guo et al. [12] a median follow-up time of 11.5 months is given. The EORTC CATNON trial provides a median follow-up of 27.0 months and the TAVAREC trial a median follow-up time of 28.0 months [42, 43].

*MGMT* status was described in different extents in all studies. Most gliomas were *MGMT*-promoter methylated in Tesileanu et al. [40] (68.0%) and Draaisma et al. [9] (73.0%). In Hsu et al. [17] the *MGMT* status is only given for a minority of patients (methylated: 25.1%, unmethylated 7.2%), Guo et al. [12] reported methylation for 35.9% of patients as methylated.

More detailed patient characteristics are displayed in Supplementary Table 1 in Additional file 1.

### Characteristics of tumor treatment

#### IDH *-wt gliomas*

Hsu et al. [17] state the resection status as positive (67.7%) or negative (32.3%). Guo et al. [12] describe the resection status as total resection (61.8%), subtotal resection (17.8%) and biopsy (20.4%).

Information on adjuvant therapy schemes is provided in Hsu et al. [17], where all (167) patients received RT of the tumor cavity (mostly 60 Gy in 30 fractions) as well as concomitant and adjuvant TMZ treatment. Likewise, postoperative treatment in Guo et al. [12] comprised the combination of RT and TMZ (75/117, 64.1%) according to the Stupp-Protocol.

#### IDH *-mut gliomas*

In Tesileanu et al. [40] the percentage of biopsies (15.5%) and resections (84.5%) is given but not further specified concerning total or subtotal resection. Draaisma et al. [9] did not report initial tumor resection status.

For postoperative treatment in the CATNON cohort of Tesileanu et al. [40] patients were equally randomized to receive RT alone (33 fractions of 1.8 Gy), RT with concurrent TMZ (75mg/m^2^ daily for 7 weeks), RT with adjuvant TMZ (150-200mg/m^2^ on day 1–5 in 12 four-week-cycles) or RT with concurrent and adjuvant TMZ. In Draaisma et al. [9] almost all patients received initial RT (97.5%) while some patients underwent prior chemotherapy containing PCV (2, 1.6%) or TMZ (26, 21.3%). At recurrence, all patients (122) were randomized and received either chemotherapy containing TMZ (61, 50%) or TMZ and BEV (61, 50%). All other studies do not report treatment types at recurrence.

More detailed treatment characteristics are displayed in Supplementary Table 1 in Additional file 1.

### Frequency of *CDKN2A/B* deletion

Overall, in all studies the frequency of homozygous *CDKN2A/B* deletion was 23.8% (170/714). There was a clear difference in prevalence of *CDKN2A/B* abnormality dependent on *IDH*-mutation status. In *IDH*-wt gliomas, more than half of the tumors (57.1%) harbored the deletion in contrast to only 9.8% of *IDH*-mut gliomas.

In all chosen studies homozygous deletion of *CDKN2A/B* was analyzed. OS data for hemizygous deletions was not available. In Hsu et al. [17] the median OS for *CDKN2A* deletion was 13.2 months and for *CDKN2B* deletion 12.8 months. In Guo et al. [12] *CDKN2A/B* was summarized and the median OS was 12.3 months. A higher median OS is depicted in Tesileanu et al. [40] with 36.6 months. In Draaisma et al. [9] the primary *IDH*-mut gliomas showed a median OS of 36.6 months.

### Survival analysis of individual patient data

#### IDH *-wt gliomas*

**Fig. 2:**
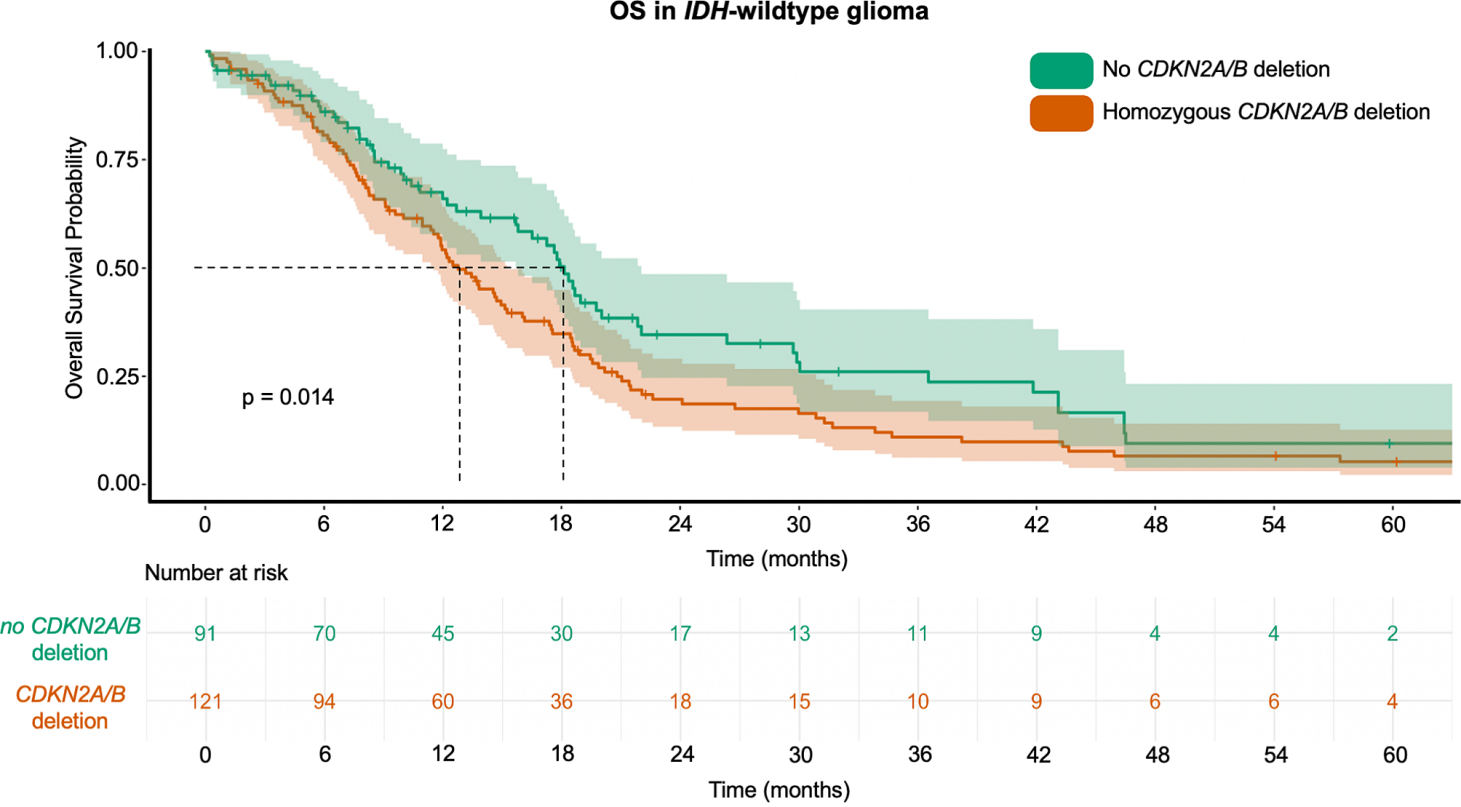
Kaplan-Meier chart of *IDH*-wt glioma displaying overall survival probability stratified by non-deleted *CDKN2A/B* (green) and *CDKN2A/B* deletion (orange)

Median survival of patients with *IDH*-wt glioma and homozygous *CDKN2A/B* deletion was 13.0 months (95%-CI 11.2–14.8). In contrast, median survival of *IDH*-wt GBM with non-deleted *CDKN2A/B* was 18.0 months (95%-CI 16.2–19.8); χ²(1) = 6.086, *p* = 0.014, Log-Rank. Therefore, a homozygous deletion of *CDKN2A/B* in glioma patients with *IDH*-wt increased risk of death, HR = 1.6 (95%-CI 1.1–2.1), Fig. 2. In the *IDH*-wt cohort the 1-year OS rate without *CDKN2A/B* deletion was 55%, with homozygous deletion it was 36%. After two years the OS was 32% and 17% respectively. Detailed OS rates from 6 to 60 months are fully displayed in Table 1.

#### IDH *-mut gliomas*

**Fig. 3:**
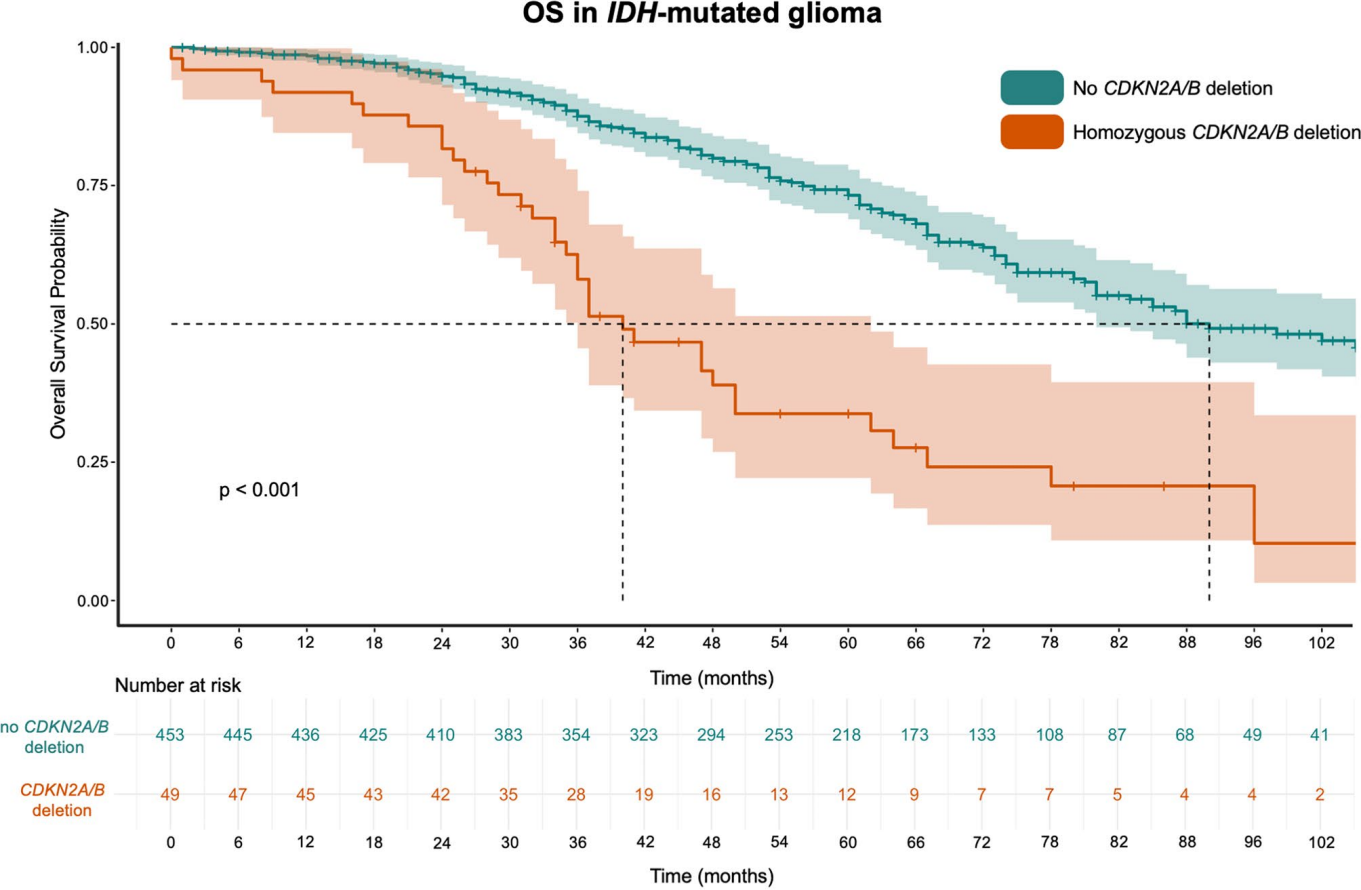
Kaplan-Meier chart of *IDH*-mut glioma displaying overall survival probability stratified by non-deleted *CDKN2A/B* (teal) and *CDKN2A/B* deletion (orange)

Median overall survival time in patients with *IDH*-mut glioma without *CDKN2A/B* deletion was 92.0 months (95%-CI 76.6–107.4) compared to 40.0 months with *CDKN2A/B* deletion (95%-CI 30.4–49.6); χ²(1) = 42.8; *p* < 0.001, Log-Rank. A *CDKN2A/B* deletion was associated with a significantly shorter overall survival time (HR = 3.2; 95%-CI 2.2–4.5), Fig. 3. The 1-year OS in the *IDH*-mut cohort was 97% and 88% respectively. After two years 92%/73% survived.

Detailed OS rates from 6 to 60 months are fully displayed in Table 1.

Likewise, OS differed greatly depending on *IDH*-mutation status. In all patients regardless of *CDKN2A/B* status the median OS time in patients with *IDH*-wt was 15.0 months (95%-CI 12.19–17.81). In contrast the median OS time with *IDH*-mutation was 87.0 months (95%-CI 77.16–96.84); χ²(1) = 29.56; *p* < 0.001, Log-Rank.

Detailed OS rates from 6 to 60 months are fully displayed in Table 1.

**Table 1:** Overall survival rates at several time points depending on *CDKN2A/B* status in *IDH*-wt and *IDH*-mut glioma

*IDH* status	time (months)	OS regarding *CDKN2A/B* status	
		no deletion	homozygous deletion
wildtype	6	67%	60%
	12	55%	36%
	18	34%	20%
	24	32%	17%
	60	15%	5%
mutated	6	99%	92%
	12	97%	88%
	18	95%	86%
	24	92%	73%
	60	69%	28%

### Predictive value of *p16* IHC staining

In 10 eligible studies a total of 1087 samples were examined. In all studies IHC was used to determine *p16* status. The cut-off for retention differed between the studies. Bortolotto et al. [4], Geyer et al. [11], Rao et al. [31] and Vij et al. [44] classified samples *p16* retained when at least 5% of the cells showed IHC staining while Park et al. [27], Purkait et al. [29] set a cut-off of 1%. In Maragkou et al. [23], Purkait et al. [30] and Suman et al. [38] a complete absence of staining was classified as *p16* loss. Burns et al. [6] did not give further explanation how specimens were classified as retained or absent but stated that four slides were stained as confirmation of the result. Information on details of cell count is missing in some studies. Bortolotto et al. [4] scanned 10 high-power fields at 400x magnification in each tumor section. Comparably Vij et al. [44] used the average of the minimum and maximum percentage of tumor cell staining in an area of highest tumor agglomeration at 10x magnification. An amount of 1000 cells were counted in Purkait et al. [29] as well in areas of strongest nuclear staining requiring at least 10 representative microscopic fields at 400x magnification. To determine *CDKN2A/B* status a Multiplex PCR was performed in Bortolotto et al. [4], Burns et al. [6] and Rao et al. [31]. Vij et al. [44] performed NGS. In all other studies fluorescence in situ hybridization (FISH) was used to define the *CDKN2A/B* status. In Geyer et al. [11] and Purkait et al. [31] the *CDKN2A/B* deletion status as hemi- or homozygous was stated separately (Geyer et al. [11]: 15 hetero-, 38 homozygous; Purkait et al. [31]: 7 hemi-, 20 homozygous, Maragkou et al. [23]: 18 hemi-, 33 homozygous). Geyer et al. [11] did not include the hemizygous samples in the results. In the following analysis a hemizygous deletion of *CDKN2A/B* was treated as a homozygous deletion.

Patient characteristics of and how *p16* IHC was evaluated in the included studies are summarized in Supplementary Tables 2 and 3 in Additional file 1.

### P16 immunohistochemistry as surrogate marker for homozygous *CDKN2A/B* deletions

Of all pooled samples in 734/1087 (68%) cases the *CDKN2A/B* gene was not deleted. Conversely, 353/1087 (32%) showed *CDKN2A/B* deletions. A total of 662/1087 samples (61%) were classified as *p16* retained and 425/1087 (40%) were determined as *p16* absent (Table 2). The correlation of *CDKN2A/B* status and *p16* staining can be summarized as following: In 588/662 *p16* retained cases *CDKN2A/B* deletion was not detected, implying a NPV of *p16* staining of 88.8%. Conversely, 279/425 *p16* absent cases showed a *CDKN2A/B* deletion resulting in a PPV of 65.6%. With these total numbers the accuracy of a *p16* staining to predict the *CDKN2A/B* status can be calculated. The cumulative data of the studies show that in 279/353 cases the *p16* staining correctly identified the deletion of *CDKN2A/B* meaning its sensitivity results in 79.0%. The specificity of absent *p16* staining to identify 588/734 samples with non-deletion of *CDKN2A/B* is 80.1%.

**Table 2:** Crosstab of association of *p16* and *CDKN2A/B* status of all pooled samples

*overall*	CDKN2A/B deletion not detected	CDKN2A/B deletion detected	
*p16* IHC retained	588 (54%)	74 (7%)	662 (61%)
*p16* IHC absent	146 (13%)	279 (26%)	425 (39%)
	734 (68%)	353 (32%)	1087 (100%)

After pooling of respective samples different cut-offs for *p16* IHC retention impacted diagnostic accuracy (Table 3, Supp. Tables 4–6).

Concerning sensitivity, the 5% cut-off (89.6% ± 5.8%) showed a higher diagnostic accuracy when compared to both the 1% cut-off (71.2% ± 7.1%) and complete absence as cut-off (77.2% ± 9.2%). For specificity, the 5% cut-off (91.3% ± 3.7%) scored highest compared to both the 1% cut-off (71.7% ± 5.7%) and complete absence as cut-off (80.0% ± 4.9%). Regarding PPV, using a 5% cut-off achieved 82.6% ± 6.9%, higher than the 1% cut-off (62.4% ± 7.1%) and complete absence as cut-off (54.5% ± 9.2%). For NPV, a difference was noted between the 5% cut-off (95.0% ± 2.9%) and the 1% cut-off (79.1% ± 5.4%), as well as between the 1% cut-off and complete absence as cut-off (91.9% ± 3.6%).

**Table 3:** Comparison of diagnostic accuracy of metrics of different cut-offs for *p16* IHC retention

Metric	Sensitivity	Specificity	PPV	NPV
5% cut-off	89.6% ± 5.8%	91.3% ± 3.7%	82.6% ± 6.9%	95.0% ± 2.9%
1% cut-off	71.2% ± 7.1%	71.7% ± 5.7%	62.4% ± 7.1%	79.1% ± 5.4%
Complete absence as cut-off	77.2% ± 9.2%	80.0% ± 4.9%	54.5% ± 9.2%	91.9% ± 3.6%

Almost all data could be separated between *IDH*-mut astrocytoma, *IDH*-wt GBM and *IDH*-mut 1p/19q codeleted ODG regarding their *p16* IHC testing and *CDKN2A/B* status. Geyer et al. [11] presented *p16* IHC data for every glioma subtype but did not show the *CDKN2A/B* status for the samples separately. Park et al. [27] did not specify *p16* status for *CDKN2A/B* status of glioma subtypes. The diagnostic accuracy of *p16* IHC testing varied between *IDH*-mut and *IDH-*wt glioma (Table 4, Supp. Tables 7–10). Sensitivity between *IDH*-mut glioma (82.0% ± 11.6%) and *IDH*-wt GBM (80.8% ± 7.0%) was comparable high. The *p16* IHC testing in *IDH*-mut glioma showed a specificity of 85.3% ± 4.2%, which appeared higher than in *IDH*-wt GBM (68.3% ± 9.1%). Similarly, NPV in *IDH*-mut glioma outperformed results in *IDH*-wt GBM (96.3% ± 2.4% vs. 74.2% ± 8.9%).

When comparing different glioma subtypes differences in diagnostic accuracy were again noted. Sensitivity in *IDH*-mut 1p/19q codeleted ODG (87.5% ± 16.2%) outperformed *IDH*-wt GBM (80.8% ± 6.9%) and *IDH*-mut astrocytoma (79.4% ± 13.6%).

Specificity was lowest in *IDH*-wt GBM (68.3% ± 9.1%) compared to *IDH*-mut astrocytoma (85.2% ± 4.6%) and *IDH*-mut 1p/19q codeleted ODG (86.0% ± 10.4%). PPV in *IDH*-wt GBM (75.9% ± 7.3%) was higher than in *IDH*-mut astrocytoma (44.3% ± 12.5%) and comparable to *IDH*-mut 1p/19q codeleted ODG (70.0% ± 20.1%). NPV was lower for *IDH*-wt GBM (74.2% ± 8.9%) when compared to both *IDH*-mut 1p/19q codeleted ODG (94.9% ± 6.9%) and *IDH*-mut astrocytoma (96.5% ± 2.5%).

**Table 4:** Comparison of *p16* IHC retention in different glioma subtypes

Metric	Sensitivity	Specificity	PPV	NPV
*IDH*-mut 1p/19q codeleted ODG	87.5% ± 16.2%	86.0% ± 10.4%	70.0% ± 20.1%	94.9% ± 6.9%
*IDH*-mut astrocytoma	79.4% ± 13.6%	85.2% ± 4.6%	44.3% ± 12.5%	96.5% ± 2.5%
*IDH*-mut glioma	82.0% ± 11.6%	85.3% ± 4.2%	50.6% ± 10.9%	96.3% ± 2.4%
*IDH*-wt GBM	80.8% ± 6.9%	68.3% ± 9.1%	75.9% ± 7.3%	74.2% ± 8.9%

### Associations between *p16* IHC status and patient survival

Survival analysis regarding *p16* IHC status only is mostly not provided in the selected studies. Park et al. [27] demonstrated significant difference in cumulative survival depending on *p16* loss in the whole glioma cohort (*p* < 0.001) and in a Multivariate Cox regression analysis *p16* IHC loss was significantly associated with worse survival in *IDH*-mut gliomas (HR = 2.6, 95%-CI 1.3–5.4, *p* = 0.008). Geyer et al. [11] presented significant difference in cumulative survival of *IDH*-wt GBM (*p* < 0.001) and grade 3 ODG (*p* < 0.001). Interestingly, the *CDKN2A* status in *p16* absent samples showed no significant difference between non-deleted and homozygous deleted samples in the whole glioma cohort (*p* = 0.97). Suman et al. [38] reported worse 3-year OS (*p16* absent 54% vs. 76% *p16* retained (*p* = 0.039) and PFS (3-year survival: *p16* absent 43% vs. 76% *p16* retained; *p* = 0.0045).

## Discussion

Recent advancements in molecular genetics have enhanced our understanding of glioma pathophysiology and clinical development, especially concerning their prognostic value and potential outcomes. The deletion of the *CDKN2A/B* gene, known to influence tumor progression and clinical outcomes, has been corroborated in other malignant central nervous system (CNS) neoplasms, including meningiomas [36, 45].

A previous meta-analysis pooled dichotomous data on *CDKN2A/B* deletions (tumor recurrence/death: yes or no) [22]. However, according to the Cochrane handbook, combining dichotomous data in conventional meta-analyses may lead to less reliable conclusions [16]. Instead, time-to-event data, especially with IPD, is recommended for more accurate meta-analyses [33]. Notably, the earlier meta-analysis by Lu et al. [22] did not use the now preferred method to reconstruct IPD. Therefore, we performed an IPD meta-analysis using longitudinal time-to-event data to more precisely assess the survival impact of *CDKN2A/B* deletions. We discovered that on average, 23.8% of gliomas have some form of *CDKN2A/B* deletion, either heterozygous or homozygous. Notably, 57.1% of *IDH*-wt GBM possess homozygous deletions, compared to only 9.8% in *IDH*-mut gliomas. These findings emphasize *CDKN2A/B*’s significant role in glioma progression and its effectiveness as a prognostic marker for OS in large *IDH*-wt and *IDH*-mut glioma cohorts.

The survival effect of intact *CDKN2A/B* in *IDH*-wt gliomas quantitatively reaches that of the survival benefit of the addition of temozolomide to radiotherapy in the “Stupp” trial in GBM (HR = 0.63) [37]. In *IDH*-mut gliomas the survival effect of intact *CDKN2A/B* resembles that of the survival benefit from post-radiation chemotherapy in low grade *IDH*-mut gliomas (HR = 0.38) in the RTOG9802 trial [3].

Homozygous deletions of *CDKN2A/B* emerge as potent biomarkers in gliomas, leading to uncontrolled cell cycle activity and increased cell proliferation [34], (Fig. S1 in Additional file 1). The *CDKN2A/B* genes encode three proteins that suppress the oncogenic cyclin-dependent kinase (CDK) pathway, with loss of function in proteins *p14*-*p16* resulting in a dysregulated cell cycle and impacting other oncogenic pathways, including angiogenesis [7, 13]. For example, *p14* inhibits endothelial cell migration by enhancing tissue inhibitor of metalloproteinase 3 expression [49], while *p16* curtails angiogenesis by regulating vascular endothelial growth factors [13]. Additionally, heterozygous *CDKN2A/B* deletions in gliomas, detected in 17% of primary *IDH*-mut gliomas, hold prognostic significance akin to homozygous deletions, highlighting their relevance in glioma progression [18]. However, the lack of IPD hindered including this finding in our pooled analysis.

Our research corroborates the significant negative impact that homozygous deletion of *CDKN2A* has on the prognosis of patients with *IDH*-mut astrocytoma. Previous recent studies have noted that a homozygous deletion of *CDKN2A/B* correlates with a particularly adverse outcome in these patients, even among those with CNS WHO grade 4 tumors [10, 19, 35, 46]. Therefore, our results reinforce the notion that this genetic alteration serves as an independent marker for CNS WHO grade 4 pathologies [21]. The current WHO classification recommends testing for *CDKN2A/B* homozygous deletion in *IDH*-mut astrocytomas that exhibit anaplastic characteristics of CNS WHO grade 3. This recommendation does not extend to *IDH*-mut ODG that are neuropathologically consistent with CNS WHO grade 2 gliomas [21], as these typically do not have the *CDKN2A/B* homozygous deletion [35].

With increasing complexity and costs there is significant need for surrogate markers for *CDKN2A/B* deletions/alterations that can easily be tested. A recent investigation of 100 *IDH*-wt and *IDH*-mut gliomas cases showed a good correlation between *p16* immunostaining and the presence of homozygous *CDKN2A* deletions across *IDH*-wt and *IDH*-mut tumors of all WHO grades. In tumors with neuropathologist-scored *p16* greater than 20%, they found 100% specificity for excluding homozygous *CDKN2A* deletions, and in tumors with *p16* equal to or less than 5%, they observed 100% specificity for predicting homozygous *CDKN2A* deletions. Hence, this study concluded that *p16* immunohistochemistry is a cost effective and convenient method for evaluating *CDKN2A* homozygous deletions in gliomas, as an alternative to expensive genomic sequencing [44].

In our pooled analysis of all available *p16* immunohistochemistry test results, these had a Youden’s Index of 0.59 and 79.0% sensitivity and 80.1% specificity for true cases of *CDKN2A* deletions. A 21% chance of errors persisted, emphasizing the need for corroborating tests and eventually standardized methods with clear cut-offs in this important field. A cut-off value of 5% demonstrated the highest diagnostic accuracy across all test parameters, especially in terms of sensitivity and PPV, to reliably identify affected patients. Regarding glioma subtype the accuracy was higher in *IDH*-mut glioma potentially explained by the increased cellular heterogeneity observed in *IDH*-wt GBM, including areas of necrosis and other structural variations. The low PPV alongside high sensitivity and NPV in *IDH*-mut glioma and astrocytoma appears due to the data distribution, with relatively high false positives reducing PPV, while low false negatives and high true negatives support high sensitivity and NPV. Where available, survival outcomes among glioma patients varied based on *p16* status. Eventually, its cost-effectiveness and accessibility might make *p16* immunohistochemistry a preferred preliminary screening tool, especially where genetic testing is less available. Further, it might be a valuable option for refined prognostic assessment in patients with *IDH*-wt GBM and an age ≥ 55 years where genetic testing is currently regarded as not-necessary according to recommendations of the WHO classification [47].

However, its ability to detect functional inactivation of *CDKN2A/B* through nonsynonymous *CDKN2A/B* mutation, which have recently been encountered in 2.6% of *IDH*-mut astrocytoma cases, or of heterozygous deletions might be reduced [15, 28]. Further investigation is needed to determine the significance of *p16* effects, not tied to *CDKN2A/B* deletion but due to alternative inactivation mechanisms, such as truncating mutations or hypermethylation.

Our results underscore the necessity for future research to implement more uniform *p16* scoring methods to increase comparability. Developing definitive guidelines for *p16* retention thresholds would enhance the reliability of *p16* IHC as a prognostic indicator for *CDKN2A/B* deletions. Against this backdrop, the present pooled results still must be interpreted with caution due to heterogeneous methodology regarding cut-off determination in *p16* IHC.

Prognostication by biomarkers is the first step to establish novel targeted therapies. However, the implications of *CDKN2A/B* deletion status regarding survival is complex in the novel stratification of gliomas by the *IDH* status.

The influence of *IDH* mutations on glioma outcomes is closely linked to the status of tumor-suppressor genes. The anti-tumor effect of *IDH* mutations is nullified by *CDKN2A/B* deletion, diminished with *TP5*3 mutations or 1p/19q codeletions, and strongest with intact tumor-suppressor genes (Fig. S2 in Additional file 1). Essentially, the presence of *CDKN2A/B* deletion marks a critical decline in prognosis for *IDH*-mut gliomas [2, 22, 35]. Without this deletion, *TP53* mutations in *IDH*-mut astrocytomas correlate with reduced survival compared to *TP5*3-wildtype [24], underscoring the role of tumor-suppressor genes in cancer progression. Conversely, in tumors with intact *TP53* and *CDKN2A*, *IDH1* mutations significantly inhibit glioma development [41]. Thus, the loss of key tumor-suppressor genes, especially *CDKN2A/B*, undermines *IDH* mutation’s suppressive effects concerning more aggressive glioma types.

The recent meta-analysis faces several limitations. The data of four clinical studies was included, which represents a limited dataset, especially with regards to analysis of potential confounding effects. However, data of 714 patients (212 *IDH*-wt GBM and 502 *IDH*-mut astrocytoma) was accumulated which exceeds the usual patient number of large clinical trials and adds significant accuracy to the effect estimations of individual studies. The included studies predate the latest WHO CNS tumor classification [21] and lacked refinement in individual patient data across crucial factors such as *MGMT* promoter methylation, resection extent, and treatment specifics. Despite this, data stratification by *IDH* status provided a substantial cohort of *IDH*-mut gliomas. Variability in resection extent among studies could affect detected deletion rates and PFS evaluations [8]. Detection methods for *CDKN2A/B* deletions vary in precision, employing techniques like SNP microarrays, NGS, methylation studies, and FISH. Integrating *CDKN2A* and *CDKN2B* analyses through NGS for targeted and whole-genome approaches, and using methylation arrays like HumanMethylation450 and MethylationEPIC, enhances diagnostic accuracy but can result in inter-lab result discrepancies. Genome-wide methylation analysis also offers significant prognostic data [26]. It is crucial to assess and compare these methodologies for their predictive accuracy in PFS and OS [48].

In conclusion, the present meta-analysis is the first investigation using reconstructed IPD to analyze the impact of homozygous *CDKN2A/B* deletions on OS in 714 *IDH*-wt or *IDH*-mut gliomas. The results prove that homozygous *CDKN2A/B* deletions are strong negative prognostic markers for OS in both *IDH*-mut and *IDH*-wt gliomas. *P16* immunohistochemistry seems to be a promising surrogate tool with varying accuracy depending on cut-offs and tumor types. The standardization of scoring and detection of other functionally relevant *CDKN2A/B* alterations needs to be further investigated. Eventually, these findings might provide information that aid future clinical studies investigating targeted drug therapies for *IDH-*mut*/IDH-*wt gliomas with *CDKN2A/B* gene alterations.

## Electronic supplementary material

Below is the link to the electronic supplementary material.

Supplementary Material 1

## Data Availability

The datasets generated during and/or analyzed during the current study are available from the corresponding author on reasonable request.

## Abbreviations

BEV: Bevacizumab
CDK: Cyclin-Dependent Kinase
CDKN2A/B: Cyclin-Dependent Kinase Inhibitor 2 A/2B
CI: Confidence Interval
CNS: Central Nervous System
FISH: Fluorescence In Situ Hybridization
GBM: Glioblastoma
HR: Hazard Ratio
IHC: Immunohistochemistry
IDH: Isocitrate Dehydrogenase
IPD: Individual Patient Data
MTAP: Methylthioadenosine Phosphorylase
NGS: Next Generation Sequencing
NPV: Negative Predictive Value
ODG: Oligodendroglioma
OS: Overall Survival
PPV: Positive Predictive Value
PFS: Progression-Free Survival
SNP: Single Nucleotide Polymorphism
TMZ: Temozolomide
WHO: World Health Organization

## References

[1] Aoki K, Nakamura H, Suzuki H, Matsuo K, Kataoka K, Shimamura T, Motomura K, Ohka F, Shiina S, Yamamoto T, Nagata Y, Yoshizato T, Mizoguchi M, Abe T, Momii Y, Muragaki Y, Watanabe R, Ito I, Sanada M, Yajima H, Morita N, Takeuchi I, Miyano S, Wakabayashi T, Ogawa S, Natsume A (2018) Prognostic relevance of genetic alterations in diffuse lower-grade gliomas. Neuro-Oncol 20:66–77. 10.1093/neuonc/nox132 29016839 10.1093/neuonc/nox132 PMC5761527

[2] Appay R, Dehais C, Maurage C-A, Alentorn A, Carpentier C, Colin C, Ducray F, Escande F, Idbaih A, Kamoun A, Marie Y, Mokhtari K, Tabouret E, Trabelsi N, Uro-Coste E, Delattre J-Y, Figarella-Branger D, Network POLA (2019) CDKN2A homozygous deletion is a strong adverse prognosis factor in diffuse malignant IDH-mutant gliomas. Neuro-Oncol noz124. 10.1093/neuonc/noz124 10.1093/neuonc/noz124 PMC7145561 31832685

[3] Bell EH, Zhang P, Shaw EG, Buckner JC, Barger GR, Bullard DE, Mehta MP, Gilbert MR, Brown PD, Stelzer KJ, McElroy JP, Fleming JL, Timmers CD, Becker AP, Salavaggione AL, Liu Z, Aldape K, Brachman DG, Gertler SZ, Murtha AD, Schultz CJ, Johnson D, Laack NN, Hunter GK, Crocker IR, Won M, Chakravarti A (2020) Comprehensive Genomic Analysis in NRG Oncology/RTOG 9802: a phase III trial of Radiation Versus Radiation Plus Procarbazine, Lomustine (CCNU), and Vincristine in High-Risk Low-Grade Glioma. J Clin Oncol 38:3407–3417. 10.1200/JCO.19.02983 32706640 10.1200/JCO.19.02983 PMC7527157

[4] Bortolotto S, Chiadò-Piat L, Cavalla P, Bosone I, Chiò A, Mauro A, Schiffer D (2000) CDKN2A/p16 inactivation in the prognosis of oligodendrogliomas. Int J Cancer 88:554–557. https://doi.org/10.1002/1097-0215(20001115)88:4<554::aid-ijc6>3.0.co;2-q 11058870 10.1002/1097-0215(20001115)88:4<554::aid-ijc6>3.0.co;2-q

[5] Brat DJ, Aldape K, Colman H, Figrarella-Branger D, Fuller GN, Giannini C, Holland EC, Jenkins RB, Kleinschmidt-DeMasters B, Komori T, Kros JM, Louis DN, McLean C, Perry A, Reifenberger G, Sarkar C, Stupp R, Van Den Bent MJ, Von Deimling A, Weller M (2020) cIMPACT-NOW update 5: recommended grading criteria and terminologies for IDH-mutant astrocytomas. Acta Neuropathol (Berl) 139:603–608. 10.1007/s00401-020-02127-9 31996992 10.1007/s00401-020-02127-9 PMC8443062

[6] Burns KL, Ueki K, Jhung SL, Koh J, Louis DN (1998) Molecular genetic correlates of p16, cdk4, and pRb immunohistochemistry in glioblastomas. J Neuropathol Exp Neurol 57:122–130. 10.1097/00005072-199802000-00003 9600204 10.1097/00005072-199802000-00003

[7] Cánepa ET, Scassa ME, Ceruti JM, Marazita MC, Carcagno AL, Sirkin PF, Ogara MF (2007) INK4 proteins, a family of mammalian CDK inhibitors with novel biological functions. IUBMB Life 59:419–426. 10.1080/15216540701488358 17654117 10.1080/15216540701488358

[8] Coons SW, Johnson PC (1993) Regional Heterogeneity in the proliferative activity of human gliomas as measured by the Ki-67 labeling index. J Neuropathol Exp Neurol 52:609–618. 10.1097/00005072-199311000-00008 8229080 10.1097/00005072-199311000-00008

[9] Draaisma K, Tesileanu CMS, de Heer I, Klein M, Smits M, Reijneveld JC, Clement PM, de Vos FYF, Wick A, Mulholland PJ, Taphoorn MJB, Weller M, Chinot OL, Kros JM, Verschuere T, Coens C, Golfinopoulos V, Gorlia T, Idbaih A, Robe PA, van den Bent MJ, French PJ (2022) Prognostic significance of DNA methylation profiles at MRI enhancing Tumor recurrence: a report from the EORTC 26091 TAVAREC trial. Clin Cancer Res off J Am Assoc Cancer Res 28:2440–2448. 10.1158/1078-0432.CCR-21-3725 10.1158/1078-0432.CCR-21-3725 35294545

[10] Fortin Ensign SP, Jenkins RB, Giannini C, Sarkaria JN, Galanis E, Kizilbash SH (2023) Translational significance of CDKN2A/B homozygous deletion in isocitrate dehydrogenase-mutant astrocytoma. Neuro-Oncol 25:28–36. 10.1093/neuonc/noac205 35973817 10.1093/neuonc/noac205 PMC9825307

[11] Geyer L, Wolf T, Chenard M-P, Cebula H, Schott R, Noel G, Guerin E, Pencreach E, Reita D, Entz-Werlé N, Lhermitte B (2023) p16 immunohistochemical expression as a Surrogate Assessment of CDKN2A Alteration in Gliomas leading to Prognostic significances. Cancers 15:1512. 10.3390/cancers15051512 36900302 10.3390/cancers15051512 PMC10000516

[12] Guo X, Gu L, Li Y, Zheng Z, Chen W, Wang Y, Wang Y, Xing H, Shi Y, Liu D, Yang T, Xia Y, Li J, Wu J, Zhang K, Liang T, Wang H, Liu Q, Jin S, Qu T, Guo S, Li H, Wang Y, Ma W (2023) Histological and molecular glioblastoma, IDH-wildtype: a real-world landscape using the 2021 WHO classification of central nervous system tumors. Front Oncol 13:1200815. 10.3389/fonc.2023.1200815 37483487 10.3389/fonc.2023.1200815 PMC10358772

[13] Harada H, Nakagawa K, Iwata S, Saito M, Kumon Y, Sakaki S, Sato K, Hamada K (1999) Restoration of wild-type p16 down-regulates vascular endothelial growth factor expression and inhibits angiogenesis in human gliomas. Cancer Res 59:3783–3789 10446996

[14] Hartmann C, Hentschel B, Wick W, Capper D, Felsberg J, Simon M, Westphal M, Schackert G, Meyermann R, Pietsch T, Reifenberger G, Weller M, Loeffler M, Von Deimling A (2010) Patients with IDH1 wild type anaplastic astrocytomas exhibit worse prognosis than IDH1-mutated glioblastomas, and IDH1 mutation status accounts for the unfavorable prognostic effect of higher age: implications for classification of gliomas. Acta Neuropathol (Berl) 120:707–718. 10.1007/s00401-010-0781-z 21088844 10.1007/s00401-010-0781-z

[15] Hickman RA, Gedvilaite E, Ptashkin R, Reiner AS, Cimera R, Nandakumar S, Price A, Vanderbilt C, Fahy T, Young RJ, Miller AM, Mellinghoff IK, Rosenblum MK, Ladanyi M, Arcila ME, Zhang Y, Brannon AR, Bale TA (2023) CDKN2A/B mutations and allele-specific alterations stratify survival outcomes in IDH-mutant astrocytomas. Acta Neuropathol (Berl) 146:845–847. 10.1007/s00401-023-02639-0 37831210 10.1007/s00401-023-02639-0 PMC10628020

[16] Higgins JP, Li T, Deeks JJ (2023) Chap. 6: Choosing effect measures and computing estimates of effect. In: Cochrane Handb. Syst. Rev. Interv. V64. https://training.cochrane.org/handbook/current/chapter-06. Accessed 22 Apr 2024

[17] Hsu EJ, Thomas J, Maher EA, Youssef M, Timmerman RD, Wardak Z, Dan TD, Patel TR, Vo DT (2022) Impact of CDKN2A/B, MTAP, and TERT genetic alterations on Survival in IDH Wild Type Glioblastomas. Discov Oncol 13:126. 10.1007/s12672-022-00590-2 36380219 10.1007/s12672-022-00590-2 PMC9666584

[18] Kocakavuk E, Johnson KC, Sabedot TS, Reinhardt HC, Noushmehr H, Verhaak RGW (2023) Hemizygous *CDKN2A* deletion confers worse survival outcomes in IDHmut-noncodel gliomas. Neuro-Oncol 25:1721–1723. 10.1093/neuonc/noad095 37329568 10.1093/neuonc/noad095 PMC10479907

[19] Korshunov A, Casalini B, Chavez L, Hielscher T, Sill M, Ryzhova M, Sharma T, Schrimpf D, Stichel D, Capper D, Reuss DE, Sturm D, Absalyamova O, Golanov A, Lambo S, Bewerunge-Hudler M, Lichter P, Herold‐Mende C, Wick W, Pfister SM, Kool M, Jones DTW, Von Deimling A, Sahm F (2019) Integrated molecular characterization of *IDH*‐mutant glioblastomas. Neuropathol Appl Neurobiol 45:108–118. 10.1111/nan.12523 30326163 10.1111/nan.12523

[20] Liu N, Zhou Y, Lee JJ (2021) IPDfromKM: reconstruct individual patient data from published Kaplan-Meier survival curves. BMC Med Res Methodol 21:111. 10.1186/s12874-021-01308-8 34074267 10.1186/s12874-021-01308-8 PMC8168323

[21] Louis DN, Perry A, Wesseling P, Brat DJ, Cree IA, Figarella-Branger D, Hawkins C, Ng HK, Pfister SM, Reifenberger G, Soffietti R, Von Deimling A, Ellison DW (2021) The 2021 WHO classification of tumors of the Central Nervous System: a summary. Neuro-Oncol 23:1231–1251. 10.1093/neuonc/noab106 34185076 10.1093/neuonc/noab106 PMC8328013

[22] Lu VM, O’Connor KP, Shah AH, Eichberg DG, Luther EM, Komotar RJ, Ivan ME (2020) The prognostic significance of CDKN2A homozygous deletion in IDH-mutant lower-grade glioma and glioblastoma: a systematic review of the contemporary literature. J Neurooncol 148:221–229. 10.1007/s11060-020-03528-2 32385699 10.1007/s11060-020-03528-2

[23] Maragkou T, Reinhard S, Jungo P, Pasquier B, Neuenschwander M, Schucht P, Vassella E, Hewer E (2023) Evaluation of MTAP and p16 immunohistochemical deficiency as surrogate marker for CDKN2A/B homozygous deletion in gliomas. Pathol (Phila) 55:466–477. 10.1016/j.pathol.2023.01.005 10.1016/j.pathol.2023.01.005 37032198

[24] Murnyak B, Huang LE (2021) Association of TP53 Alteration with tissue specificity and patient outcome of IDH1-Mutant glioma. Cells 10:2116. 10.3390/cells10082116 34440884 10.3390/cells10082116 PMC8394030

[25] Ostrom QT, Price M, Neff C, Cioffi G, Waite KA, Kruchko C, Barnholtz-Sloan JS (2022) CBTRUS Statistical Report: primary brain and other Central Nervous System tumors diagnosed in the United States in 2015–2019. Neuro-Oncol 24:v1–v95. 10.1093/neuonc/noac202 36196752 10.1093/neuonc/noac202 PMC9533228

[26] Ozair A, Bhat V, Alisch RS, Khosla AA, Kotecha RR, Odia Y, McDermott MW, Ahluwalia MS (2023) DNA methylation and histone modification in low-Grade gliomas: current understanding and potential clinical targets. Cancers 15:1342. 10.3390/cancers15041342 36831683 10.3390/cancers15041342 PMC9954183

[27] Park JW, Kang J, Lim KY, Kim H, Kim S-I, Won JK, Park C-K, Park S-H (2021) The prognostic significance of p16 expression pattern in diffuse gliomas. J Pathol Transl Med 55:102–111. 10.4132/jptm.2020.10.22 33348944 10.4132/jptm.2020.10.22 PMC7987518

[28] Perry A, Anderl K, Borell TJ, Kimmel DW, Wang CH, O’Fallon JR, Feuerstein BG, Scheithauer BW, Jenkins RB (1999) Detection of p 16, RB, CDK4, and p53 gene deletion and amplification by fluorescence in situ hybridization in 96 gliomas. Am J Clin Pathol 112:801–809. 10.1093/ajcp/112.6.801 10587703 10.1093/ajcp/112.6.801

[29] Purkait S, Jha P, Sharma MC, Suri V, Sharma M, Kale SS, Sarkar C (2013) CDKN2A deletion in pediatric versus adult glioblastomas and predictive value of p16 immunohistochemistry. Neuropathol off J Jpn Soc Neuropathol 33:405–412. 10.1111/neup.12014 10.1111/neup.12014 23311918

[30] Purkait S, Sharma V, Jha P, Sharma MC, Suri V, Suri A, Sharma BS, Sarkar C (2015) EZH2 expression in gliomas: correlation with CDKN2A gene deletion/ p16 loss and MIB-1 proliferation index. Neuropathol off J Jpn Soc Neuropathol 35:421–431. 10.1111/neup.12201 10.1111/neup.12201 26096306

[31] Rao LS, Miller DC, Newcomb EW (1997) Correlative immunohistochemistry and molecular genetic study of the inactivation of the p16INK4A genes in astrocytomas. Diagn Mol Pathol Am J Surg Pathol Part B 6:115–122. 10.1097/00019606-199704000-00008 10.1097/00019606-199704000-00008 9098651

[32] Reis GF, Pekmezci M, Hansen HM, Rice T, Marshall RE, Molinaro AM, Phillips JJ, Vogel H, Wiencke JK, Wrensch MR, Walsh KM, Perry A (2015) CDKN2A loss is associated with shortened overall survival in lower-grade (World Health Organization Grades II-III) astrocytomas. J Neuropathol Exp Neurol 74:442–452. 10.1097/NEN.0000000000000188 25853694 10.1097/NEN.0000000000000188 PMC4397174

[33] Riley RD, Lambert PC, Abo-Zaid G (2010) Meta-analysis of individual participant data: rationale, conduct, and reporting. BMJ 340:c221–c221. 10.1136/bmj.c221 20139215 10.1136/bmj.c221

[34] Sharpless NE (2005) INK4a/ARF: a multifunctional tumor suppressor locus. Mutat Res 576:22–38. 10.1016/j.mrfmmm.2004.08.021 15878778 10.1016/j.mrfmmm.2004.08.021

[35] Shirahata M, Ono T, Stichel D, Schrimpf D, Reuss DE, Sahm F, Koelsche C, Wefers A, Reinhardt A, Huang K, Sievers P, Shimizu H, Nanjo H, Kobayashi Y, Miyake Y, Suzuki T, Adachi J-I, Mishima K, Sasaki A, Nishikawa R, Bewerunge-Hudler M, Ryzhova M, Absalyamova O, Golanov A, Sinn P, Platten M, Jungk C, Winkler F, Wick A, Hänggi D, Unterberg A, Pfister SM, Jones DTW, van den Bent M, Hegi M, French P, Baumert BG, Stupp R, Gorlia T, Weller M, Capper D, Korshunov A, Herold-Mende C, Wick W, Louis DN, von Deimling A (2018) Novel, improved grading system(s) for IDH-mutant astrocytic gliomas. Acta Neuropathol (Berl) 136:153–166. 10.1007/s00401-018-1849-4 29687258 10.1007/s00401-018-1849-4

[36] Sievers P, Hielscher T, Schrimpf D, Stichel D, Reuss DE, Berghoff AS, Neidert MC, Wirsching H-G, Mawrin C, Ketter R, Paulus W, Reifenberger G, Lamszus K, Westphal M, Etminan N, Ratliff M, Herold-Mende C, Pfister SM, Jones DTW, Weller M, Harter PN, Wick W, Preusser M, von Deimling A, Sahm F (2020) CDKN2A/B homozygous deletion is associated with early recurrence in meningiomas. Acta Neuropathol (Berl) 140:409–413. 10.1007/s00401-020-02188-w 32642869 10.1007/s00401-020-02188-w PMC7423850

[37] Stupp R, Mason WP, Van Den Bent MJ, Weller M, Fisher B, Taphoorn MJB, Belanger K, Brandes AA, Marosi C, Bogdahn U, Curschmann J, Janzer RC, Ludwin SK, Gorlia T, Allgeier A, Lacombe D, Cairncross JG, Eisenhauer E, Mirimanoff RO (2005) Radiotherapy plus Concomitant and Adjuvant Temozolomide for Glioblastoma. N Engl J Med 352:987–996. 10.1056/NEJMoa043330 15758009 10.1056/NEJMoa043330

[38] Suman S, Sharma R, Katiyar V, Mahajan S, Suri A, Sharma MC, Sarkar C, Suri V (2022) Role of CDKN2A deletion in grade 2/3 IDH-mutant astrocytomas: need for selective approach in resource-constrained settings. Neurosurg Focus 53:E17. 10.3171/2022.9.FOCUS22427 36455270 10.3171/2022.9.FOCUS22427

[39] Sun Y, Sun Y, Yan K, Li Z, Xu C, Geng Y, Pan C, Chen X, Zhang L, Xi Q (2019) Potent anti-tumor efficacy of palbociclib in treatment-naïve H3.3K27M-mutant diffuse intrinsic pontine glioma. EBioMedicine 43:171–179. 10.1016/j.ebiom.2019.04.043 31060906 10.1016/j.ebiom.2019.04.043 PMC6558223

[40] Tesileanu CMS, van den Bent MJ, Sanson M, Wick W, Brandes AA, Clement PM, Erridge SC, Vogelbaum MA, Nowak AK, Baurain JF, Mason WP, Wheeler H, Chinot OL, Gill S, Griffin M, Rogers L, Taal W, Rudà R, Weller M, McBain C, van Linde ME, Sabedot TS, Hoogstrate Y, von Deimling A, de Heer I, van IJcken WFJ, Brouwer RWW, Aldape K, Jenkins RB, Dubbink HJ, Kros JM, Wesseling P, Cheung KJ, Golfinopoulos V, Baumert BG, Gorlia T, Noushmehr H, French PJ (2021) Prognostic significance of genome-wide DNA methylation profiles within the randomized, phase 3, EORTC CATNON trial on non-1p/19q deleted anaplastic glioma. Neuro-Oncol 23:1547–1559. 10.1093/neuonc/noab088 33914057 10.1093/neuonc/noab088 PMC8408862

[41] Tiburcio PDB, Xiao B, Chai Y, Asper S, Tripp SR, Gillespie DL, Jensen RL, Huang LE (2018) IDH1R132H is intrinsically tumor-suppressive but functionally attenuated by the glutamate-rich cerebral environment. Oncotarget 9:35100–35113. 10.18632/oncotarget.26203 30416682 10.18632/oncotarget.26203 PMC6205547

[42] Van Den Bent MJ, Baumert B, Erridge SC, Vogelbaum MA, Nowak AK, Sanson M, Brandes AA, Clement PM, Baurain JF, Mason WP, Wheeler H, Chinot OL, Gill S, Griffin M, Brachman DG, Taal W, Rudà R, Weller M, McBain C, Reijneveld J, Enting RH, Weber DC, Lesimple T, Clenton S, Gijtenbeek A, Pascoe S, Herrlinger U, Hau P, Dhermain F, Van Heuvel I, Stupp R, Aldape K, Jenkins RB, Dubbink HJ, Dinjens WNM, Wesseling P, Nuyens S, Golfinopoulos V, Gorlia T, Wick W, Kros JM (2017) Interim results from the CATNON trial (EORTC study 26053 – 22054) of treatment with concurrent and adjuvant temozolomide for 1p/19q non-co-deleted anaplastic glioma: a phase 3, randomised, open-label intergroup study. Lancet 390:1645–1653. 10.1016/S0140-6736(17)31442-3 28801186 10.1016/S0140-6736(17)31442-3 PMC5806535

[43] Van Den Bent MJ, Klein M, Smits M, Reijneveld JC, French PJ, Clement P, De Vos FYF, Wick A, Mulholland PJ, Taphoorn MJB, Lewis J, Weller M, Chinot OL, Kros JM, De Heer I, Verschuere T, Coens C, Golfinopoulos V, Gorlia T, Idbaih A (2018) Bevacizumab and temozolomide in patients with first recurrence of WHO grade II and III glioma, without 1p/19q co-deletion (TAVAREC): a randomised controlled phase 2 EORTC trial. Lancet Oncol 19:1170–1179. 10.1016/S1470-2045(18)30362-0 30115593 10.1016/S1470-2045(18)30362-0

[44] Vij M, Cho BB, Yokoda RT, Rashidipour O, Umphlett M, Richardson TE, Tsankova NM (2023) P16 immunohistochemistry is a sensitive and specific surrogate marker for CDKN2A homozygous deletion in gliomas. Acta Neuropathol Commun 11:73. 10.1186/s40478-023-01573-2 37138345 10.1186/s40478-023-01573-2 PMC10155323

[45] Wach J, Basaran AE, Arlt F, Vychopen M, Seidel C, Barrantes-Freer A, Müller W, Gaunitz F, Güresir E (2023) CDKN2A/B deletions are strongly associated with meningioma progression: a meta-analysis of individual patient data. Acta Neuropathol Commun 11:189. 10.1186/s40478-023-01690-y 38017560 10.1186/s40478-023-01690-y PMC10685484

[46] Weller M, Felsberg J, Hentschel B, Gramatzki D, Kubon N, Wolter M, Reusche M, Roth P, Krex D, Herrlinger U, Westphal M, Tonn JC, Regli L, Maurage C-A, Von Deimling A, Pietsch T, Le Rhun E, Reifenberger G (2024) Improved prognostic stratification of patients with isocitrate dehydrogenase-mutant astrocytoma. Acta Neuropathol (Berl) 147:11. 10.1007/s00401-023-02662-1 38183430 10.1007/s00401-023-02662-1 PMC10771615

[47] WHO Classification of Tumours Editorial Board (2021) World health organization classification of tumours of the central nervous system tumours, 5th edn. International Agency for Research on Cancer, Lyon

[48] Yuile A, Satgunaseelan L, Wei JQ, Rodriguez M, Back M, Pavlakis N, Hudson A, Kastelan M, Wheeler HR, Lee A (2023) CDKN2A/B homozygous deletions in Astrocytomas: A literature review. Curr Issues Mol Biol 45:5276–5292. 10.3390/cimb45070335 37504251 10.3390/cimb45070335 PMC10378679

[49] Zerrouqi A, Pyrzynska B, Febbraio M, Brat DJ, Van Meir EG (2012) P14ARF inhibits human glioblastoma–induced angiogenesis by upregulating the expression of TIMP3. J Clin Invest 122:1283–1295. 10.1172/JCI38596 22378045 10.1172/JCI38596 PMC3314443

