# Supplementary material for: Homozygous CDKN2A/B deletions in low- and high-grade glioma: a meta-analysis of individual patient data and predictive values of p16 immunohistochemistry testing
Source: Acta Neuropathol Commun. 2024 Nov 26;12:180. doi: 10.1186/s40478-024-01889-7 (PMC11590270; doi:10.1186/s40478-024-01889-7)
Supplement: Supplementary file 1 — Supplementary Material 1 [file 40478_2024_1889_MOESM1_ESM.pdf]

	Glioblastoma (*IDH*-wt)		Glioma Grade 2-4 (*IDH*-mut)	
	**Hsu et al. (2022)**	**Guo et al. (2023)**	**Draaisma et al. (2022)**	**Tesileanu et al. (2021)**
**Total n (n included)**	167 (167)	191 (45)	122 (70)	654 (432)
**Histology**				
Astrocytoma	–	45 (23.6%)[1]	84 (68.6%)	654 (100%)
Glioblastoma	167 (100%)	146 (76.4%)	18 (14.8%)[2]	–
Oligodendroglioma	–	–	3 (2.5%)[1]	–
Inconculsive	–	–	17 (13.9%)	–
***CDKN2A/B* status determined by**	NGS	NGS	DNA methylation profiling	DNA methylation profiling
**Sex**				
Female	66 (39.5%)	91 (47.6%)	42 (34.4%)	271 (41.4%)
Male	101 (60.5%)	100 (52.4%)	80 (65.6%)	383 (58.6%)
**Age**				
Range	24.8-85.1	55.5 ± 14.7	43 (34, 52)	18-82
Median	63.9	–	–	41
**WHO grading**				
2	–	18 (9.4%)	29 (23.8%)	–
3	–	27 (14.1%)	44 (36.1%)	654 (100%)
4	167 (100%)	146 (76.4%)	29 (23.8%)	–
**Karnofsky Index**		Median 80		
>80	92 (55.1%)	–	50 (41.0%)	384 (58.7%)
<80	75 (44.9%)	–	72 (59.0%)	268 (41.0%)
**WHO performance status**				
0	–	–	50 (41.0%)	384 (58.7%)
1	–	–	61 (50.0%)	268 (41.0%)[3]
2	–	–	11 (9.0%)	
**Extent of resection**				
Total resection				
Positive	113 (67.7%)	118 (61.8%)	–	540 (82.6%)[4]
Negative	14 (8.4%)	–	–	–
Subtotal resection	–	–	–	–
Biopsy	–	39 (20.4%)	–	114 (17.4%)
***MGMT* status**				
Unmethylated	12 (7.2%)	93/145 (64.1%)	11 (15.7%)	204 (31.2%)
Methylated	42 (25.1%)	52/145 (35.9%)	59 (84.3%)	445 (68.0%)
Missing	113 (67.7%)	–	–	10 (1.5%)
**Initial post OP treatment**		–	–	
RT alone	–	–	119 (97.5%)	168 (25.7%)
RT+concurrent & adjuvant TMZ	167 (100%)	–	–	165 (25.2%)
RT+concurrent TMZ	–	–	–	162 (24.8%)
RT+adjuvant TMZ	–	–	–	159 (24.3%)
Missing	–	42/117 (35.8 %)	3 (2.5%)	–
**Treatment at recurrence**				
TMZ	–	–	61 (50.0%)	–
TMZ+BV	–	–	61 (50.0%)	–

Supplementary Table 1: Patient characteristics of included studies analyzing OS and CDKN2A/B status

[1] all cases were classified as molecular glioblastoma
[2] not included in data analysis
[3] WHO performance status >0
[4] not specified if total or subtotal resection

	Bortolotto et al. (2000)	Burns et al. (1998)	Geyer et al. (2023)	Maragkou et al. (2023)	Park et al. (2021)	Purkait et al. (2013)	Purkait et al. (2015)	Rao et al. (1997)	Suman et al. (2022)	Vij et al. (2023)
**Total n (n included)**	25 (25)	25 (25)	173 (173)	100 (100)	326 (326)	67 (67)	108 (84)	37 (37)	150 (150)	100 (100)
**Histology**										
Oligodendro-glioma	25 (100%)	–	27 (16%)	5 (5%)	30 (9.2%)	–	21 (19.4%)	–	–	8 (8%)
Astrocytoma	–	–	54 (31%)	38 (38%)	101 (31%)	–	87 (80.6%)	36 (100%)	150 (100%)	25 (25%)
Glioblastoma	–	25 (100%)	63 (36%)	41 (41%)	195 (59.8%)	67 (100%)	–	–	–	62 (62%)
Other	–	–	29 (17%)	16 (16%)	–	–	–	–	–	5 (5%)
**Age**										
Range	–	–	1–79	1–88	16–82	5–72	19–72	3–80	21–70	2–85
Mean	–	–	–	–	–	–	–	–	II: 32.9<br>III: 42.2	–
Median	–	–	36.5	47.5	54	–	–	42	–	54
***CDKN2A/B* status**										
Detected via	Multiplex PCR	Multiplex PCR	FISH	FISH	FISH	FISH	FISH	Multiplex PCR	FISH	NGS
Wildtype	16 (64%)	13 (52%)	120 (69%)	49 (49%)	197 (60.4%)	40 (60%)	62 (57.4%)	26 (70%)	144 (96%)	52 (52%)
Homozygous deletion	9 (36%)	12 (48%)	38 (22%)	33 (33%)	129 (39.6%)	20 (30%)	22 (20.4%)	11 (30%)	6 (4%)	48 (48%)
Heterozygous deletion	–	–	15 (9%)	18 (18%)	–	7 (10%)	–	–	–	–
***p16* IHC**										
Retained	15 (60%)	5 (20%)	117 (68%)	65 (65%)	183 (56.1%)	32 (48%)	41 (38%)	26 (70%)	116 (77%)	62 (62%)
Absent	10 (40%)	20 (80%)	56 (32%)	35 (35%)	143 (43.9%)	35 (52%)	43 (40%)	11 (30%)	34 (23%)	38 (38%)
**WHO grading**										
1	–	–	49 (28%)	13 (13%)	–	–	–	–	–	6 (6%)
2	12 (48%)	–	14 (8%)	14 (14%)	23 (7%)	–	26 (24.1%)	15 (%)	95 (63.3%)	8 (8%)
3	13 (52%)	–	23 (13%)	17 (17%)	70 (22%)	–	27 (25%)	31 (%)	49 (32.7%)	15 (15%)
4	–	25 (100%)	87 (50%)	55 (55%)	233 (71%)	67 (100%)	55 (50.9%)	–	6 (4%)	68 (68%)
Not attributed	–	–	–	–	–	–	–	–	–	3 (3%)
***IDH* status**										
Wildtype	–	–	71 (41%)	47 (46%)	223 (68.4%)	–	–	–	–	71 (71%)
Mutated	–	–	42 (24%)	29 (29%)	103 (22.4%)	–	–	–	150 (100%)	29 (29%)
Not given	–	–	60 (35%)	24 (24%)	–	–	–	–	–	–

Supplementary Table 2: Summary of patient characteristics of the included studies for evaluation of *p16* immunohistochemistry staining

	Used *p16* clone and staining conditions	Labeling index Cut-off and further required conditions	Evaluation via
**Bortolotto et al. (2000)**	G175-405<br><br>FFPE sections, peroxidase deactivation (3% H_2O_2), blocking step (10% normal rabbit serum) → AB diluted 1:1500 in TBS buffer, incubation overnight at 4°C, streptavidin-biotin complex method with metal-enhanced DAB	>5%<br><br>nuclear staining	visual inspection (10 high-power fields at x400 in all areas of each tumor section)
**Burns et al. (1998)**	JC8<br><br>FFPE sections , peroxidase deactivation (0.5% H_2O_2) in methanol, antigen retrieval in citrate buffer, blocked in normal horse serum/milk → primary AB at 1:500 for 2 hrs, then biotinylated secondary AB for 1 hour, avidin-biotin complex kit for 1 hour, DAB staining, HE counterstain	–<br><br>4 copies of each specimen evaluated	visual inspection
**Geyer et al. (2023)**	E6H4<br><br>–	>5%<br><br>both nuclear and cytoplasmatic staining; 3 copies of each specimen evaluated	visual inspection
**Maragkou et al. (2023)**	E6H4<br><br>dilution 1:5; EDTA buffer for antigen retrieval	negative = complete abscence of staining in tumor cells<br><br>–	visual inspection
**Park et al. (2021)**	E6H4<br><br>*Ventana BenchMark XT* automated IHC staining system	>1%<br><br>staining in nucleus only or concurrent nuclear and cytoplasma staining	visual inspection
**Purkait et al. (2013)**	E6H4<br><br>FFPE sections, antigen retrieval in citrate buffer, peroxidase deactivation (3% H_2O_2) in methanol, primary AB overnight incubation at 4°C, then biotin-labelled secondary antibody for 60min, strept-HRP for 30min, developed with DAB for 10min, HE counterstain	>1%<br><br>nuclear staining	visual inspection
**Purkait et al. (2015)**	–<br><br>FFPE sections, antigen retrieval in citrate buffer, primary AB overnight incubation at 4°C, universal-labeled streptavidin-biotin kit, DAB staining, HE counterstain	negative = complete abscence of staining in tumor cells<br><br>nuclear staining; 1000 cells were counted in high density areas of positive cells	visual inspection (10 microscopic fields at x400 magnification) by two independent blinded observers
**Rao et al. (1997)**	C-20<br><br>FFPE sections, antigen retrieval with pepsin, peroxidase deactivation (0.2% H_2O_2) in methanol, primary AB at 1:100, PAP method, DAB staining, HE counterstain	>5% (estimation)<br><br>nuclear staining	visual inspection
**Suman et al. (2022)**	E6H4<br><br>–	negative = complete abscence of staining in tumor cells<br><br>staining in nucleus only or concurrent nuclear and cytoplasma staining	visual inspection
**Vij et al. (2023)**	E6H4<br><br>FFPE sections, antigen retrieval in CC1 buffer, *UltraView DAB detection kit*	>5% (average of minimum and maximum)<br><br>staining in nucleus only or concurrent nuclear and cytoplasma staining in dense tumor areas	visual inspection by two pathologists (10 microscopic fields); blinded & unblinded evaluation & digital quantification via *QuPath* in the same microscopic fields

Supplementary Table 3: Summary of evaluation of *p16* immunohistochemistry staining in the included studies

<u>5% cut-off</u>	***CDKN2A/B*** **deletion not detected**	***CDKN2A/B*** **deletion detected**	
p16 **IHC retained**	209 (62%)	11 (3%)	220 (66%)
p16 **IHC absent**	20 (6%)	95 (28%)	115 (34%)
	229 (68%)	106 (32%)	335 (100%)

Supplementary Table 4: Crosstab of association of *p16* and *CDKN2A/B* status of pooled samples with a 5% cut-off

<u>1% cut-off</u>	***CDKN2A/B*** **deletion not detected**	***CDKN2A/B*** **deletion detected**	
p16 **IHC retained**	170 (43%)	45 (11%)	215 (55%)
p16 **IHC absent**	67 (17%)	111 (28%)	178 (45%)
	237 (60%)	156 (40%)	393 (100%)

Supplementary Table 5: Crosstab of association of *p16* and *CDKN2A/B* status of pooled samples with a 1% cut-off

<u>complete absence</u>	***CDKN2A/B*** **deletion not detected**	***CDKN2A/B*** **deletion detected**	
p16 **IHC retained**	204 (61%)	18 (5%)	222 (66%)
p16 **IHC absent**	51 (15%)	61 (18%)	112 (34%)
	255 (76%)	79 (24%)	334 (100%)

Supplementary Table 6: Crosstab of association of *p16* and *CDKN2A/B* status of pooled samples with a complete absence of *p16* IHC staining as cut-off

<u>*IDH*-mut glioma</u>	***CDKN2A/B*** **deletion not detected**	***CDKN2A/B*** **deletion detected**	
p16 **IHC retained**	232 (72%)	9 (3%)	241 (75%)
p16 **IHC absent**	40 (12%)	41 (13%)	81 (25%)
	272 (84%)	50 (16%)	322 (100%)

Supplementary Table 7: Crosstab of association of *p16* and *CDKN2A/B* status of *IDH*-mut glioma only

<u>*IDH*-wt GBM</u>	***CDKN2A/B*** **deletion not detected**	***CDKN2A/B*** **deletion detected**	
p16 **IHC retained**	69 (31%)	24 (11%)	93 (41%)
p16 **IHC absent**	32 (14%)	101 (45%)	133 (59%)
	101 (45%)	125 (55%)	226 (100%)

Supplementary Table 8: Crosstab of association of *p16* and *CDKN2A/B* status of *IDH*-wt GBM

<u>*IDH*-mut 1p/19q codeleted ODG</u>	***CDKN2A/B*** **deletion not detected**	***CDKN2A/B*** **deletion detected**	
p16 **IHC retained**	37 (63%)	2 (3%)	39 (66%)
p16 **IHC absent**	6 (10%)	14 (24%)	20 (34%)
	43 (73%)	16 (27%)	59 (100%)

Supplementary Table 9: Crosstab of association of *p16* and *CDKN2A/B* status of *IDH*-mut 1p/19q codeleted ODG only

<u>*IDH*-mut astrocytoma</u>	***CDKN2A/B*** **deletion not detected**	***CDKN2A/B*** **deletion detected**	
p16 **IHC retained**	195 (74%)	7 (3%)	202 (77%)
p16 **IHC absent**	34 (13%)	27 (10%)	61 (23%)
	229 (87%)	34 (13%)	263 (100%)

Supplementary Table 10: Crosstab of association of *p16* and *CDKN2A/B* status of *IDH*-mut astrocytoma only

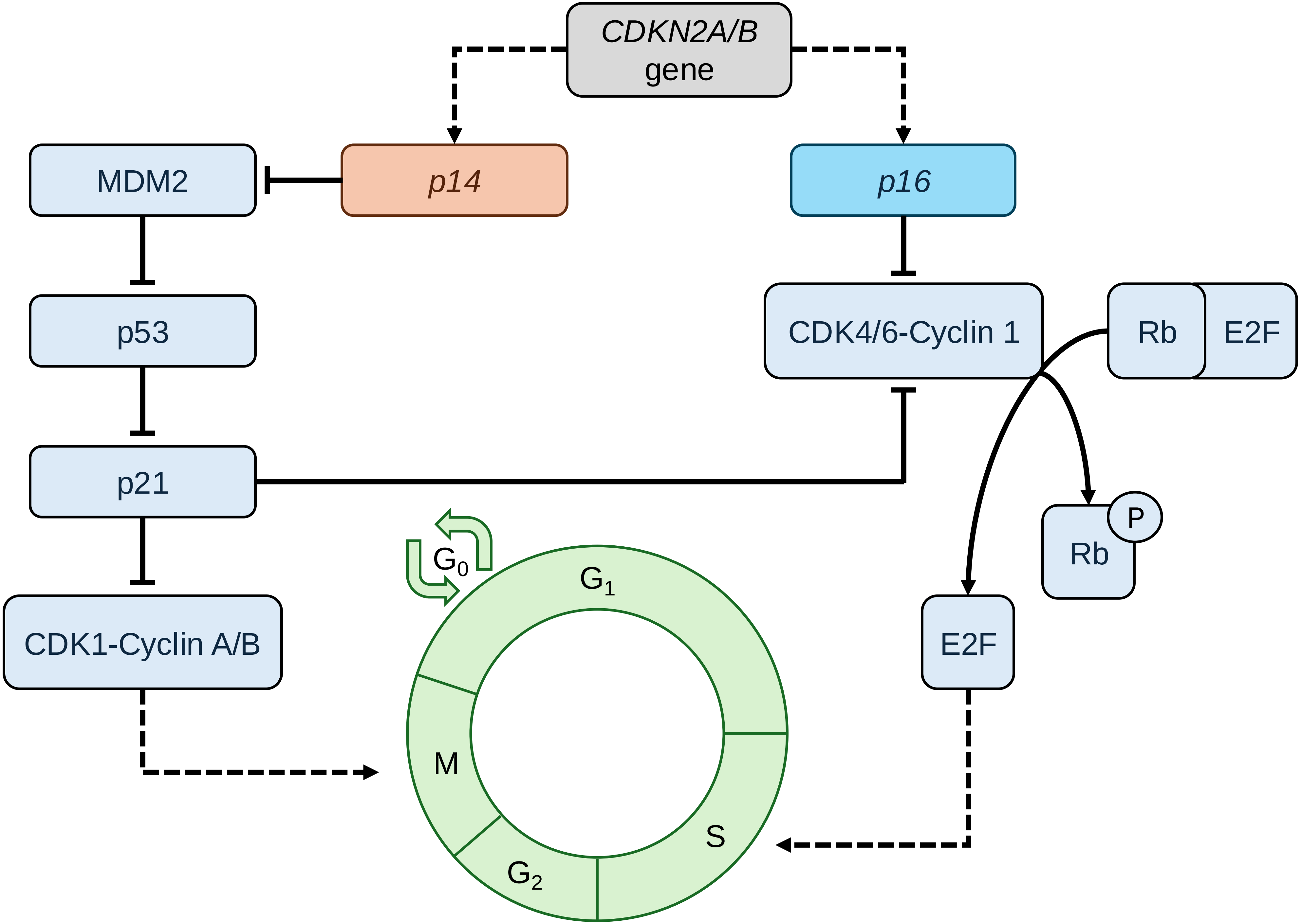

Fig. S1: *CDKN2A/B* pathway showing its gene products *p14* and *p16* suppressing the oncogenic cyclin-dependent kinase (CDK) pathway leading to a dysregulated cell cycle

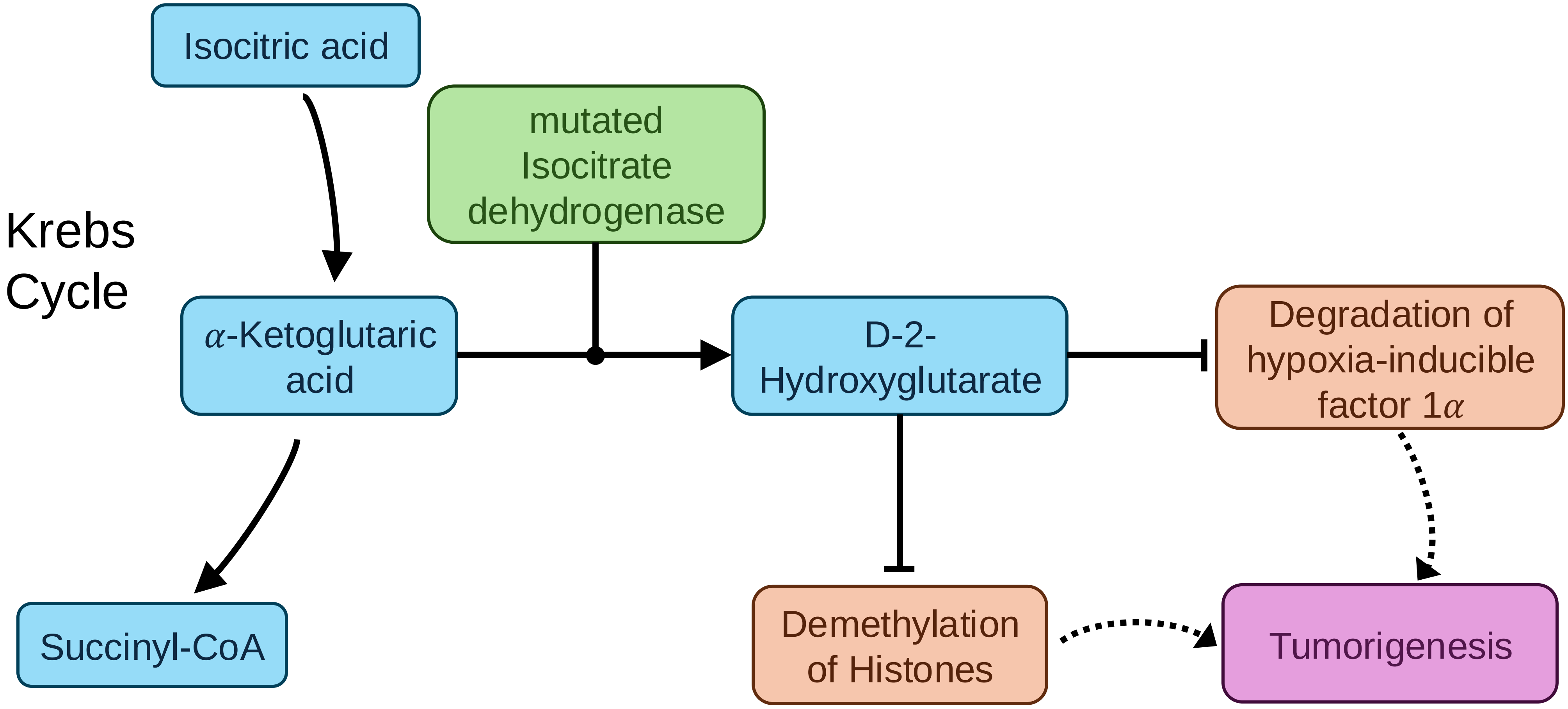

Fig. S2: Pathway of mutated isocitrate dehydrogenase generating D-2-hydroxyglutarate disrupting histone demethylation and inhibiting HIF-1α degradation as factors leading to tumorigenesis
